# Comparative analysis of antibody responses to outer surface protein (Osp)A and OspC in dogs vaccinated with Lyme disease vaccines

**DOI:** 10.1016/j.tvjl.2021.105676

**Published:** 2021-04-14

**Authors:** A.C. Camire, A.L. Hatke, V.L. King, J. Millership, D.M. Ritter, N. Sobell, A. Weber, R.T. Marconi

**Affiliations:** a Department of Microbiology and Immunology, Virginia Commonwealth University Medical Center, 1112 East Clay Street, McGuire Hall Room 101, Richmond, VA 23298-0678, USA; b Zoetis Inc., 333 Portage Road, Kalamazoo, MI 49007-4931, USA

**Keywords:** *Borrelia*, *Borreliella*, Chimeritope, *Ixodes scapularis*, Tick-borne diseases

## Abstract

Lyme disease (LD), the most common tick-borne disease of canines and humans in N. America, is caused by the spirochete *Borreliella burgdorferi*. Subunit and bacterin vaccines are available for the prevention of LD in dogs. LD bacterin vaccines, which are comprised of cell lysates of two strains of *B. burgdorferi*, contain over 1000 different proteins and cellular constituents. In contrast, subunit vaccines are defined in composition and consist of either outer surface protein (Osp)A or OspA and an OspC chimeritope. In this study, we comparatively assessed antibody responses to OspA and OspC induced by vaccination with all canine bacterin and subunit LD vaccines that are commercially available in North America.

Dogs were administered a two-dose series of the vaccine to which they were assigned (3 weeks apart): Subunit-AC, Subunit-A, Bacterin-1, and Bacterin-2. Antibody titers to OspA and OspC were determined by ELISA and the ability of each vaccine to elicit antibodies that recognize diverse OspC proteins (referred to as OspC types) assessed by immunoblot. While all of the vaccines elicited similar OspA antibody responses, only Subunit-AC triggered a robust and broadly cross-reactive antibody response to divergent OspC proteins. The data presented within provide new information regarding vaccination-induced antibody responses to key tick and mammalian phase antigens by both subunit and bacterin LD canine vaccine formulations.

## Introduction

Lyme disease (LD) is a significant health concern that affects companion animals (Littman et al., 2018) and humans (Burgdorfer et al., 1982; Benach et al., 1983). In North America, *Borreliella burgdorferi* is the primary causative agent of LD; in Europe, *B. burgdorferi*, *Borreliella bavariensis*, *Borreliella garinii*, and *Borreliella afzelii* are associated with disease (Adeolu and Gupta, 2014; Eisen, 2020). The LD spirochetes are transmitted amongst animals by *Ixodes* spp. ticks (Steere et al., 1978; Burgdorfer et al., 1982). LD is the most prevalent tick-borne disease in North America and Europe. It has been reported by the CDC that there are approximately 476,000 clinician-diagnosed cases of LD each year in humans (Kugeler et al., 2021). In dogs, 398,392 positive *B. burgdorferi* antibody (Ab) tests were catalogued by the Companion Animal Parasite Council (CAPC) in 2020 in the US.^1^ The actual number of *B. burgdorferi*-positive antibody tests is assumed to be much higher since only 30% of test data are collected by CAPC each year. The precise number of antibody-positive tests in European dogs is more difficult to determine due to differences in data collection. While a positive antibody test does not in all cases indicate active infection, it is clear from recent studies that geographic distribution of ixodid ticks is expanding (Eisen et al., 2016) and the risk of LD is increasing across the northern hemisphere (Sykes and Makiello, 2017; Vandekerckhove et al., 2019; Kugeler et al., 2021).

Clinical manifestations of canine LD are initially non-descript and, in most cases, develop slowly (Krupka and Straubinger, 2010; Little et al., 2010). Intermittent lameness due to polyarthritis is common (Levy and Magnarelli, 1992) and chronic infection can result in cardiac conduction disorders (Levy and Duray, 1988), neurological complications (Lesca et al., 2002), and protein-losing glomerulopathy leading to renal failure (Dambach et al., 1997). In experimentally-infected dogs, histological changes have been demonstrated even in dogs with sub-clinical LD. Inflammation of the tissues and joint capsules in *B. burgdorferi* infected dogs is common (Straubinger et al., 1998). Hyperkeratosis, lymphoplas-macytic vasculitis, arteritis, perineuritis, and meningitis may also develop.

Preventative strategies for LD in dogs include vaccination and the use of tick repellants and acaricides (Littman et al., 2018). In North America, subunit and bacterin LD vaccines are available (reviewed in Izac and Marconi, 2019). Subunit vaccines are defined in their composition and consist of recombinant lipidated outer surface protein A (OspA) or recombinant non-lipidated OspA in combination with a multivalent outer surface protein C (OspC) epitope-based recombinant protein (reviewed in O’Bier et al., 2020) referred to as a chimeritope (Izac et al., 2020b). In contrast to the defined antigenic composition of subunit vaccines, LD bacterin vaccines consist of more than 1000 different proteins, the overwhelming majority of which have not been demonstrated to elicit protective antibody (reviewed in O’Bier et al., 2020). All LD bacterin vaccines available in North America are a mix of cell lysates of two laboratory strains of *B. burgdorferi* (discussed in detail below).

Since the discovery of the causative agents of LD (Benach et al., 1983), OspA and OspC have been among the most intensively studied outer surface proteins produced by these pathogens. OspA and OspC are produced during distinctly different stages of the enzootic cycle (Schwan and Piesman, 2000; Schwan, 2003). OspC production is initiated within the tick midgut upon exposure to a bloodmeal and remains highly expressed during early stage infection in mammals (Caimano et al., 2019). In mammals OspC is one of the most abundant LD spirochete surface antigens (Iyer et al., 2015; Caimano et al., 2019) and it is required for the LD spirochetes to establish infection (Tilly et al., 2007; Earnhart et al., 2010). OspA is produced at high levels by spirochetes residing within the midguts of unfed ticks but it is not produced after the LD spirochetes enter into a mammal (Iyer et al., 2015; Caimano et al., 2019). While OspA plays an essential role in spirochete survival in unfed ticks, gene deletion studies have demonstrated that it is not required for survival in mammals (Pal et al., 2000). Consistent with the stages of infection during which each outer surface protein is expressed, anti-OspC antibodies can target LD spirochetes infecting both ticks and mammals, whilst anti-OspA antibodies only target LD spirochetes in the tick. The combined use of OspA and OspC as vaccine antigens (Marconi et al., 2020) elicits antibody responses that can target LD spirochetes during both stage of their enzootic cycle providing two independent and synergistic mechanisms of protection.

While the specific antigens in bacterin vaccines that contribute to protective immunity have not been defined, it has been suggested that OspA and OspC are key contributors (LaFleur et al., 2009). Due to the high level of OspA expression by laboratory-cultured LD spirochetes (Oliver Jr et al., 2016), it is likely that the OspA present in bacterin formulations elicits antibody that specifically targets spirochetes in ticks (Fikrig et al., 1992). In contrast to OspA, the low-level expression of OspC in laboratory cultured strains suggests that, in the context of a bacterin formulation, its contribution to inducing protective immunity is minimal (O’Bier et al., 2020). In addition, the expression of OspC during cultivation is limited to a subset of cells in the population (Oliver Jr et al., 2016; Xiang et al., 2017). OspC is genetically and antigenically diverse among LD isolates (Lagal et al., 2002; Earnhart and Marconi, 2007c). Distinct variants of OspC have been delineated and are referred to as OspC “types” with each assigned a letter or isolate of origin designation (OspC type A, OspC type B, OspC type PHoe, etc.) (Lagal et al., 2003; Brisson and Dykhuizen, 2004; Earnhart and Marconi, 2007c). It has been demonstrated in mice, rats, rabbits, canids (domestic and wild), horses, humans, and non-human primates that antibody responses to OspC during infection are OspC type-specific (Earnhart et al., 2005; Buckles et al., 2006; Izac et al., 2019, 2020a; Oliver Jr et al., 2016). Vaccination with a single OspC protein does not elicit production of antibodies that recognize diverse OspC types (Oliver Jr et al., 2016; Izac and Marconi, 2019), providing protection only against strains expressing closely related OspC types (Bockenstedt et al., 1997). OspC is a single copy, plasmid-encoded gene; therefore, an individual LD strain produces only a single OspC type (Marconi et al., 1993; Sadziene et al., 1993). The low-level production of OspC during cultivation and the type-specific antibody responses that it elicits raise questions regarding its relative contribution to immune responses elicited by LD bacterin vaccines.

In this study, we compared antibody responses to OspA and OspC in dogs vaccinated with the subunit and bacterin-based canine LD vaccines available in North America. In addition to antigen specific IgG titer determination, we assessed the potential of each vaccine to induce antibodies that recognize diverse OspC types and therefore potentially target diverse strains of the LD spirochetes. We demonstrate that there are both quantitative and qualitative differences in the antibody responses to OspC elicited by each vaccine. This study addresses key questions surrounding the potential contributions of OspA and OspC to protective immunity induced by administration of canine LD vaccines.

## Materials and methods

### Study inclusion/exclusion criteria

Purpose-bred beagles (21 males and 19 females; 8–9 weeks of age) were obtained from Ridglan Farms and acclimated for 7 days prior to initiating the study. Animals were sorted by date of birth and litter (dam) to form blocks of four dogs. Half of the blocks were randomly assigned to each of two rooms. Within rooms, blocks were randomly assigned to pens. The randomization was performed using a SAS program that utilizes a random number generator function (ranuni). Animals were observed at least once daily for general health and potential adverse health events. Dogs were maintained at research sites in accordance with USDA Animal Welfare Regulations (Code of Federal Regulations, Chapter 1, Subchapter A – Animal Welfare). The Zoetis Institutional Animal Care and Use Committee (IACUC) approved all protocols (Approval number, AUP # KZ-3081d-2015-06-mtw; Approval date, June 2015). Inclusion criteria consisted of negative antibody tests for OspA, OspC, and VlsE (C6 peptide) and good overall health. Screening for antibodies to OspA and OspC was done by ELISA as detailed below. To screen for antibodies to VlsE the SNAP 4Dx Plus lateral flow test was employed (IDEXX). Vaccines Subunit-AC (VANGUARD crLyme, Zoetis), Bacterin-1 (NOBIVAC Lyme, Merck), Subunit-A (RECOMBITEK Lyme, Boehringer-Ingelheim), and Bacterin-2 (Duramune Lyme, Elanco US) were randomly assigned to each treatment group and administered per the manufacturers’ label instructions on days 0 and 21. Blood was collected and serum harvested on Days 0, 21, and 35 using standard protocols.

### Ligase independent cloning (LIC) and production of recombinant proteins

The genes encoding 23 full-length OspC proteins (indicated in Fig. 2) were PCR amplified from strains of known *ospC* genotype with PCR primers that possess tail sequences that allow for ligase independent cloning (LIC). PCR was performed using *Pfu* DNA polymerase according to the manufacturers protocol (Promega). The amplicons were purified using QIAquick PCR purification kits (QIAgen), annealed with pET46 Ek/LIC or pET45b+ (Novagen), transformed into *Escherichia coli* NovaBlue(DE3) cells (Novagen), recovered, purified, and transformed into *E. coli* BL21(DE3) cells (Novagen). Protein expression was induced with IPTG (1 mM) using standard methods. The cells were recovered by centrifugation (5000 × *g*; 15 min; 4 °C), suspended in lysis buffer (50 mM NaH_2_PO_4_; 300 mM NaCl; 40 mM imidazole; lysozyme, 1 mg/mL; 30 min), sonicated, and centrifuged (15,500 × *g*; 30 min; 4 °C). The N-terminal hexahistidine tagged OspC proteins were purified from the soluble fraction by nickel affinity chromatography using a Fast Protein Liquid Chromatography system (ÄKTA; Cytiva) with a 1 mL HisTrap FF column (Cytvia). Samples were loaded into a 10 ml Superloop (Cytvia) in running buffer (50 mM NaH_2_PO_4_; 300 mM NaCl; 40 mM imidazole) followed by washing with 10 mL of running buffer. Proteins were eluted with elution buffer (50 mM NaH_2_PO_4_; 300 mM NaCl; 500 mM imidazole). One mL fractions were collected from under the peak and dialyzed into phosphate buffered saline (PBS) overnight using Spectra/Por 1 (6–8 kDa cutoff) dialysis membranes (Spectrum Laboratories). Purified OspA and the OspC chimeritope, Ch14, that are the antigens contained in the Subunit-AC vaccine, were provided by Zoetis. The concentrations of the recombinant proteins were determined using the BCA assay.

### Anti-OspA and OspC IgG titer determination

IgG titers to OspA and OspC were determined by ELISA using recombinant proteins as the immobilized antigens (250 ng per well; 96 well plates; 0.01 M borate buffer; overnight; 4 °C). Recombinant serotype 1 OspA, the most dominant OspA serotype in North America (Wilske et al., 1993), served as the detection antigen for anti-OspA antibodies and the OspC chimeritope, Ch14 (Marconi et al., 2020), served as the detection antigen for antibodies to OspC. Non-specific antibody binding was blocked by washing with blocking buffer (1% casein in PBS with 0.1% Tween 20; 300 μL per well). Primary sera, serially diluted in blocking buffer, were added to the ELISA plate wells (100 μL per well). The assay positive control for OspA and Ch14 was initially diluted to 1:25,600 and 1:6400, respectively. Plates were read when the initial dilution of the positive control reached an optical density of 1.6–2.1 at 405/490 nm. The assay negative control preimmune sera was diluted 1:200. For endpoint titer determination the serum samples were serially diluted. The minimum start dilutions were 1:25,600 and 1:6400 for OspA and Ch14, respectively. The plates were incubated (1 h; 37 °C) then horseradish peroxidase conjugated goat anti-dog IgG (H + L Chains) (Pierce) was added (1:20,000 dilution), the plates were washed and peroxidase substrate (ABTS, Sigma-Aldrich) was added (room temperature; 10–15 min). The plates were read as above and the test sample titers calculated from the average plus three standard deviations of the optical density values of negative control preimmune sera. All assays were done in duplicate.

### Sodium dodecyl sulfate polyacrylamide gel electrophoresis (SDS-PAGE) and immunoblot analyses

The recombinant OspC types (500 ng) were assessed by SDS-PAGE, visualized by staining with Coomassie brilliant blue, immunoblotted, and screened as previously described with sera collected on Day 0 and 35 at a 1:1000 dilution (Izac et al., 2019). Due to limited serum volumes, the three serum samples from each treatment group that had the highest anti-OspC IgG titers were pooled and used to screen the immunoblots of recombinant OspC types. IgG binding was detected using horseradish peroxidase-conjugated rabbit anti-dog IgG secondary antibody (1:40,000) and chemiluminescence (Clarity Western ECL; Biorad). Images were captured using the ChemiDoc imaging system (Biorad). All blots were imaged together for 148 s using the auto-optimize function. Images were cropped to remove blank spaces in order to generate a multi-panel figure.

### Statistical analyses

Antibody titers were logarithmically transformed. The transformed titers were analyzed with a general linear-mixed model for repeated measures. Pairwise treatment comparisons were made at each time point. Least square means at each time point, standard errors, and 95% confidence intervals were back-transformed to obtain the geometric mean titers (GMTs), standard errors, and their confidence intervals. In addition, minimums and maximums were calculated for each treatment and time point. All analyses were performed using the SAS software suite and all hypothesis tests were carried out at the 0.05 level of significance (two-sided, *P* < 0.05).

## Results

### Analysis of vaccination-induced antibody responses to OspA

**Fig. 1.**
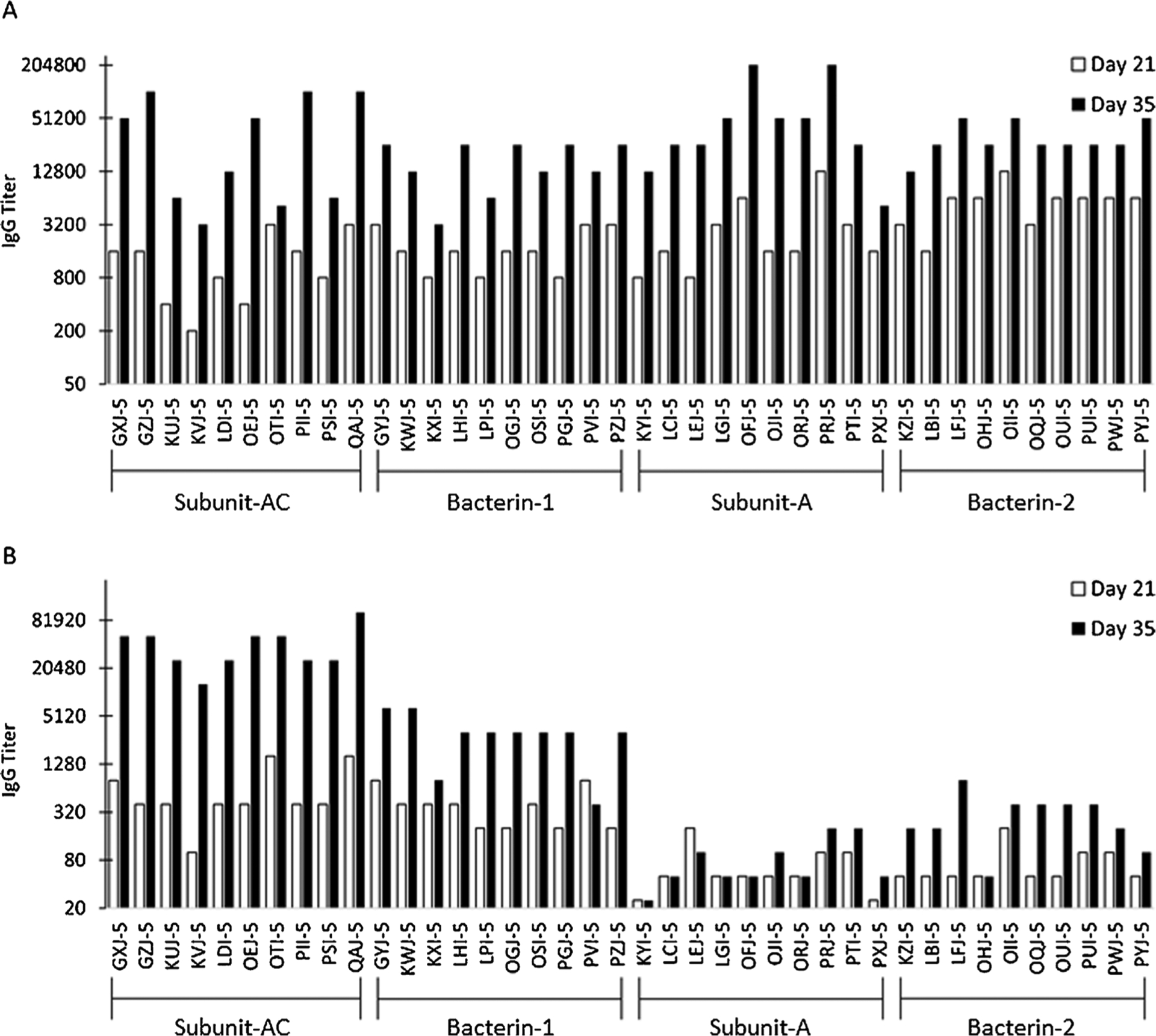
Comparative analysis of vaccination induced antibody titers to outer surface protein (Osp) A and OspC. Dogs were vaccinated with Subunit-AC, Bacterin-1, Subunit-A, and Bacterin-2. Antigen-specific IgG titers were determined in duplicate for each individual dog. Individual dog identifiers are indicated along the x axis. Panels A and B present antigen specific IgG titers for OspA (serotype 1) and OspC (Ch14), respectively.

To compare OspA antibody responses elicited by each canine LD vaccine, serum harvested from vaccinated purposed bred dogs were screened by ELISA and antigen specific IgG titers were determined for each individual animal and for each treatment group. All individual dogs, regardless of the vaccine administered, developed a robust antibody response to OspA by 2 weeks post-administration of the second vaccine dose (Day 35 sera; Fig. 1; Table 1). Differences in the OspA GMTs were noted between the Bacterin-1 and Bacterin-2 study groups. All other differences were not significant (Table 3).

### Analysis of vaccination-induced antibody responses to OspC

To compare OspC antibody responses elicited by each vaccine, the sera were screened by ELISA and individual titers and GMTS for each study group were determined. The antibody response to OspC differed among the study groups (Fig. 1; Table 2). Dogs vaccinated with Subunit-AC developed a high titer OspC-directed antibody response (GMT = 36,204) whereas the OspC GMTs for Bacterin-1 and Bacterin-2 were 2599 and 246, respectively. As expected, Subunit-A, which lacks OspC, did not elicit an anti-OspC antibody response. The difference in the GMT of study group Subunit-AC versus all other study groups at Day 35 was significant (*P* < 0.0001). Similarly, the difference in the GMT of study group Bacterin-1 versus study groups Subunit-A and Bacterin-2 was also significant (*P* = 0.0002). Table 3 presents significance of treatment pairwise comparisons of OspA and OspC antibody titers at each timepoint in the study.

### Analysis of the ability of vaccination induced anti-OspC antibody to bind to diverse OspC type proteins

**Fig. 2.**
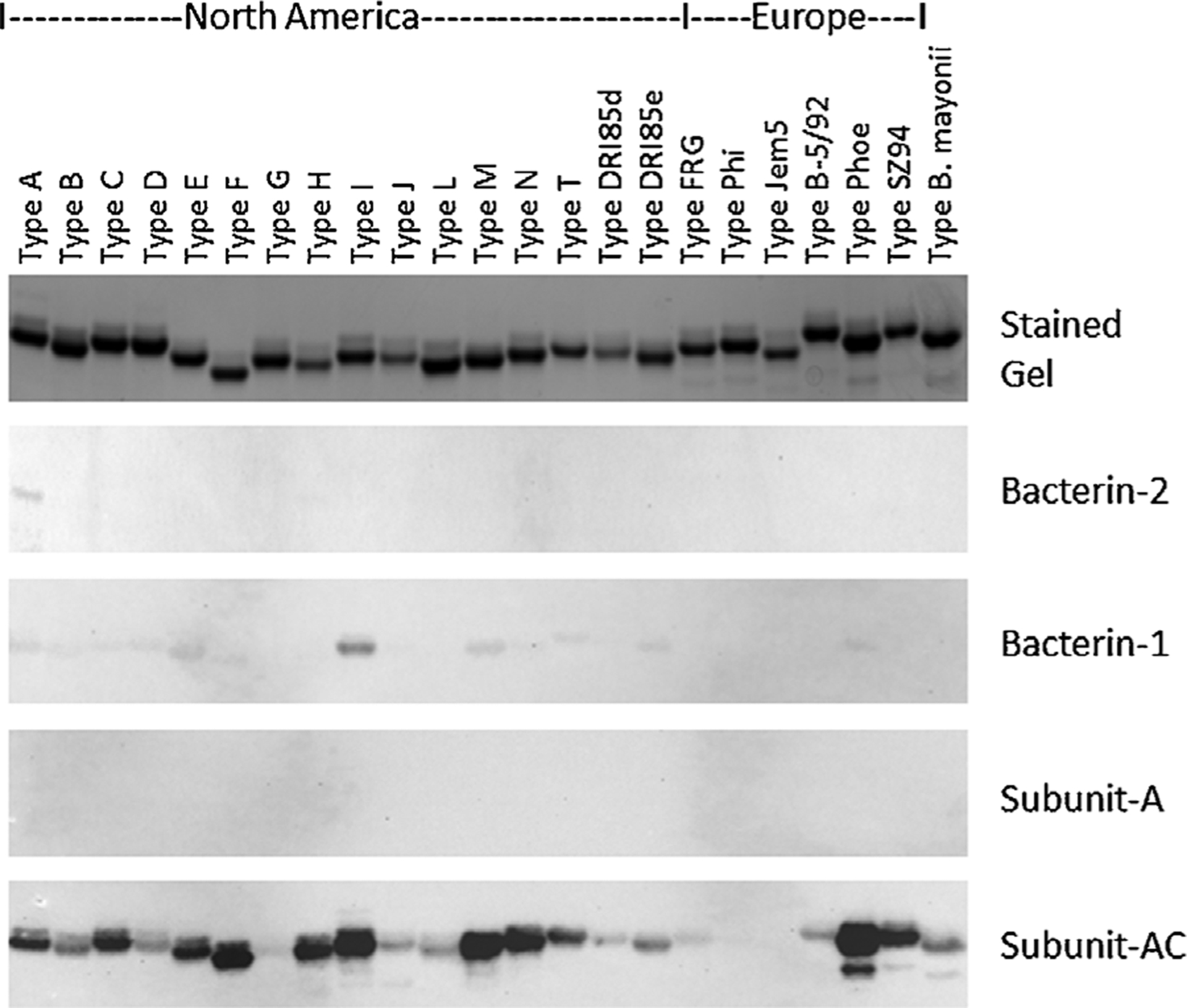
Comparative immunoblot analysis of outer surface protein (Osp) C antibody specificity. Twenty-three different OspC type proteins derived from North American or European Lyme disease isolates (as indicated) were generated as His-tagged recombinant proteins and purified using Fast Protein Liquid Chromatography. The recombinant proteins were subjected to sodium dodecyl sulfate polyacrylamide gel electrophoresis. One gel was stained to visualize the proteins and others were immunoblotted. The blots were screened with pooled sera collected from three dogs within each vaccine group 2 weeks after the second vaccine dose on Day 35. The blots were imaged together for the same amount of time (148 s). The images were cropped to generate the figure.

To assess the breadth or conversely the specificity of the IgG response to OspC in dogs administered each vaccine, pooled sera from each treatment group were screened against 23 different recombinant OspC type proteins using an immunoblot format (Fig. 2). Consistent with the high anti-OspC IgG titers elicited by Subunit-AC, sera from this study group reacted strongly with diverse OspC proteins (Fig. 2). Sera from Bacterin-1 and Bacterin-2 study groups displayed weak binding to OspC with preferential binding to types I and A, respectively (Fig. 2).

## Discussion

In this study, antibody responses to OspA and OspC in dogs administered commercially available bacterin and subunit LD vaccines were compared. After completion of the vaccine series, the anti-OspA GMTs were robust and similar for all vaccines assessed. An independent study, similarly reported that Subunit-AC and Subunit-A vaccines elicited equivalent anti-OspA antibody titers in dogs after the administration of two doses (Grosenbaugh et al., 2018). However, the anti-OspC GMTs differed significantly between vaccines. The highest OspC antibody titers were associated with Subunit-AC vaccine. The OspC antibody titers elicited by Bacterin-1 and Bacterin-2 were orders of magnitude lower (10–12x) than Subunit-AC. The low levels of OspC antibody induced by these bacterin vaccines is consistent with previous studies that have demonstrated low levels of OspC expression by *B. burgdorferi* during its cultivation (Oliver Jr et al., 2016; Xiang et al., 2017). One study reported that only 10% of the cells in a laboratory-cultured *B. burgdorferi* B31 type strain population express detectable levels of OspC (Oliver Jr et al., 2016). However, early studies detailing the development of Bacterin-1 indicate that the vaccine is derived from one *B. burgdorferi* strain that expresses OspA and a second strain that, because it is OspA-deficient (Rousselle et al., 1998), expresses increased levels of OspC (LaFleur et al., 2009). While the expression of OspA and OspC have been reported to be inversely regulated (Schwan, 2003), to our knowledge direct evidence for enhanced OspC expression by the OspA-deficient strain included in Bacterin-1 relative to any other strain has not been published. The OspC antibody titers induced by Bacterin-1 were 12 times lower than the titers induced by Subunit-AC, but the Bacterin-1 titers were 10 times higher than that induced by Bacterin-2. The identities of the strains used to formulate Bacterin-2 have not been reported and to our knowledge, the levels of OspA and OspC production by the strains that comprise this vaccine, have not been published.

Serum from dogs administered Subunit-AC was strongly immunoreactive with a diverse array of recombinant OspC proteins including OspC types associated with strains from both North America and Europe. In addition, the antibodies induced by Subunit-AC also bound to the OspC protein from the recently identified species *Borrelia mayonii* (Pritt et al., 2016a,b), suggesting a potential for cross-protection. The broad immunoreactivity of sera from dogs and other mammals vaccinated with OspC chimeritope proteins is consistent with their polyvalent epitope composition (Earnhart et al., 2007; Earnhart and Marconi, 2007a, b, c; Izac et al., 2020b). The LD bacterin vaccines assessed here elicited lower IgG titers to OspC with minimal cross reactivity with diverse OspC types. The ability of a LD vaccine to elicit robust antibody responses to both OspA and OspC is important in view of differential spirochete expression of these proteins in ticks and ticks/mammals, respectively. Since antibody targeting OspA can only bind to spirochetes within the tick mid-gut (Fikrig et al., 1992), protective efficacy of OspA alone vaccines (e.g. Subunit-A) is dependent on circulating antibody titer. While the minimal protective OspA antibody titer has not been determined in dogs, in humans it is 1200 U/mL (reviewed in O’Bier et al., 2020). Because an anamnestic response to OspA is not triggered by natural exposure to *B. burgdorferi*, repeated vaccine administration is required to maintain the minimal protective titer. The high OspA and OspC antibody titers induced by Subunit-AC allow for antibody-mediated killing to occur in both the tick and mammal with the potential for an OspC-induced anamnestic response (Hatke et al., 2020). In summary, this study provides important new information that contributes to our understanding of the antibody responses to OspA and OspC elicited by available canine LD vaccines.

## Figures and Tables

**Table 1:** Vaccination-induced anti-OspA antibody titers.

Group: Vaccine treatment	Day	Back-transformed LSM^a^	Back-transformed SE	Range	Back-transformed 95% CI
Subunit-AC	0	62	23	13–200	29–130
	21	985	236	200–3200	605–1602
	35	27,437	7668	3200–102,400	15,553–48,404
Bacterin-1	0	71	26	50–200	34–149
	21	1600	383	800–3200	983–2.603
	35	14,703	4109	3200–25,600	8334–25,939
Subunit-A	0	87	32	13–400	41–184
	21	22,263	542	800–12,800	1391–3681
	35	47,771	13,350	12,800–204,800	27,079–84,277
Bacterin-2	0	62	23	13–400	29–130
	21	5198	1244	1600–12,800	3195–8458
	35	29,407	8218	12,800–51,200	16,669–51,879

Osp, outer surface protein; LSM, least squares mean; SE, standard error; 95% CI, 95% confidence intervals.

a Due to the standard errors, the differences in OspA specific antibody titers between any vaccine were not significant.

**Table 2:** Vaccine-induced anti-OspC antibody titers.

Group: Vaccine treatment	Day	Back-trans-formed LSM	Back-transformed SE	Range	Back-transformed 95% CI
Subunit-AC	0	33	8	13–200	21–52
	21	493	111	100–1600	314–772
	35	36,205	8189	12,800–102,400	23,102–56,735
Bacterin-1	0	54	12	25–200	34–84
	21	348	79	200–800	222–546
	35	2599	588	400–6400	1659–4073
Subunit-A	0	38	9	13–200	24–59
	21	57	13	25–200	37–90
	35	71	16	25–200	45–111
Bacterin-2	0	33	8	13–100	21–52
	21	66	15	50–200	42–103
	35	246	56	50–800	157–386

Osp, outer surface protein; LSM, least squares mean; SE, standard error; 95% CI, 95% confidence intervals.

**Table 3:** Statistical significance of treatment pairwise comparisons of outer surface protein (Osp) A and OspC antibody titers.

Comparison	Day	OspA antibody titer *P*	OspC antibody titer *P*
Subunit-AC vs. Bacterin-1	0	0.7855	0.1327
	21	0.1330	0.2814
	35	0.1079	<0.0001
Subunit-AC vs. Subunit-A	0	0.4975	0.6657
	21	0.0130	<0.0001
	35	0.1508	<0.0001
Subunit-AC vs. Bacterin-2	0	1.0000	1.0000
	21	<0.0001	<0.0001
	35	0.8545	<0.0001
Bacterin-1 vs. Subunit-A	0	0.6833	0.2814
	21	0.2785	<0.0001
	35	0.0042	<0.0001
Bacterin-1 vs. Bacterin-2	0	0.7855	0.1327
	21	0.0008	<0.0001
	35	0.0758	<0.0001
Subunit-A vs. Bacterin-2	0	0.4975	0.6657
	21	0.0130	0.6657
	35	0.2065	0.0002

## Footnotes

1 See: Companion Animal Parasite Council, Parasite Prevalence Maps. https://capcvet.org/maps/#2019/all/lyme-disease/dog/united-states/ (accessed 6 April 2021)
